# Co-infection of Dengue and Malaria Complicated With Acute Respiratory Distress Syndrome (ARDS) in a Critically Ill Patient: A Case Report From a Tertiary Care Center in Jharkhand, India

**DOI:** 10.7759/cureus.108904

**Published:** 2026-05-15

**Authors:** Mahajyoti Chakravorty, Kirti Bhushan, Smita Singh, Asif Ahmed

**Affiliations:** 1 Critical Care Medicine, Tata Main Hospital, Jamshedpur, IND; 2 Anesthesiology, Tata Main Hospital, Jamshedpur, IND

**Keywords:** ards, co-infection, dengue, fluid management, malaria, mechanical ventilation, multiorgan failure

## Abstract

Co-infections of dengue and malaria are uncommon but clinically significant due to overlapping presentations and divergent management strategies. This article presents a case report of a 25-year-old adult female patient admitted to the critical care unit (CCU) of a tertiary care center in Jharkhand, India, with co-infection of *Plasmodium falciparum* malaria and dengue virus. The patient exhibited varying degrees of multiorgan failure, including severe respiratory failure necessitating mechanical ventilation, hepatic dysfunction, and hematological abnormalities. The diagnosis of dengue was established by a positive dengue nonstructural protein 1 (NS1) antigen and immunoglobulin M (IgM) antibody by enzyme-linked immunosorbent assay (ELISA), in addition to the confirmed malaria diagnosis by peripheral blood smear. The patient subsequently developed acute respiratory distress syndrome (ARDS), requiring intubation, lung-protective mechanical ventilation, and prone positioning. Management required a multidisciplinary approach involving fluid balance guided by dynamic parameters, including the inferior vena cava (IVC) distensibility index (dIVC) and serial lactate measurements, pharmacotherapy of malaria, and ventilatory support. Despite the critical nature of illness, the patient recovered with aggressive supportive care, emphasizing the importance of early recognition and individualized management in co-infection scenarios.

## Introduction

Malaria and dengue fever, as monoinfections, are among the most common vector-borne tropical febrile illnesses globally [1]. Jharkhand, an eastern state of India, is hyperendemic for malaria and endemic for dengue, with a rising number of reported cases annually [2,3]. Co-infections are frequently underreported due to overlapping clinical presentations, including fever, headache, myalgia, arthralgia, vomiting, and abdominal pain, and shared laboratory findings such as thrombocytopenia. In resource-limited settings, co-infections are often misinterpreted as monoinfections, where the first pathogen detected becomes the final diagnosis, as sequential testing incurs additional cost. Malaria is typically diagnosed by peripheral blood smear microscopy, whereas dengue diagnosis is confirmed by NS1 antigen and IgM antibody detection by enzyme-linked immunosorbent assay (ELISA).

From a critical care perspective, dengue-malaria co-infection presents unique and overlapping management challenges. The two pathogens can synergistically precipitate severe complications, including hemodynamic shock with capillary leak syndrome, worsening thrombocytopenia, hepatic dysfunction, acute kidney injury (AKI), and acute respiratory distress syndrome (ARDS), each of which may require conflicting management strategies, particularly with regard to fluid administration, vasopressor support, and ventilatory decisions. A clinical study conducted by Vasava et al. in western India between 2015 and 2020 found that 3.47% of patients with malaria also had concurrent dengue infections [4]. A similar study in eastern India between 2013 and 2016 reported a dengue-malaria co-infection rate of 3.0% [5].

This case report presents a 25-year-old female patient with dengue-malaria co-infection complicated by severe ARDS, with the aim of highlighting the importance of diagnostic vigilance, including dual pathogen testing to avoid anchoring bias, and demonstrating an evidence-based multidisciplinary approach to critical care management in this underrecognized but potentially life-threatening condition.

## Case presentation

A 25-year-old female patient with no known comorbidities presented to the emergency department (ED) with a 10-day history of high-grade fever with myalgia and rigors (maximum recorded temperature: 103°F), associated with dry cough, breathlessness, reddish discoloration of urine, diffuse abdominal pain, and vomiting over the preceding two days. She had attended a local hospital where *Plasmodium falciparum *malaria was diagnosed on the basis of ring forms on peripheral blood smear and was subsequently referred to our center. On arrival, she was conscious and alert but febrile, icteric, pale, and dyspneic. Vital signs are as follows: pulse, 130 beats/minute; BP 116/90 mmHg; respiratory rate, 24 breaths/minute; and SpO₂, 84% on room air (improving to 94% on 2-3 L/minute supplemental oxygen).

She was initially managed in the general ward with intravenous artesunate (2.4 mg/kg at 0, 12, and 24 hours, then every 24 hours) plus oral doxycycline 100 mg twice daily, supplemental oxygen, intravenous crystalloids, and supportive measures. Despite more than five days of antimalarial therapy, fever and thrombocytopenia persisted without clinical improvement. Given the highly co-endemic nature of the region for both pathogens, co-infection was suspected, and dengue serology was sent.

**Figure 1:**
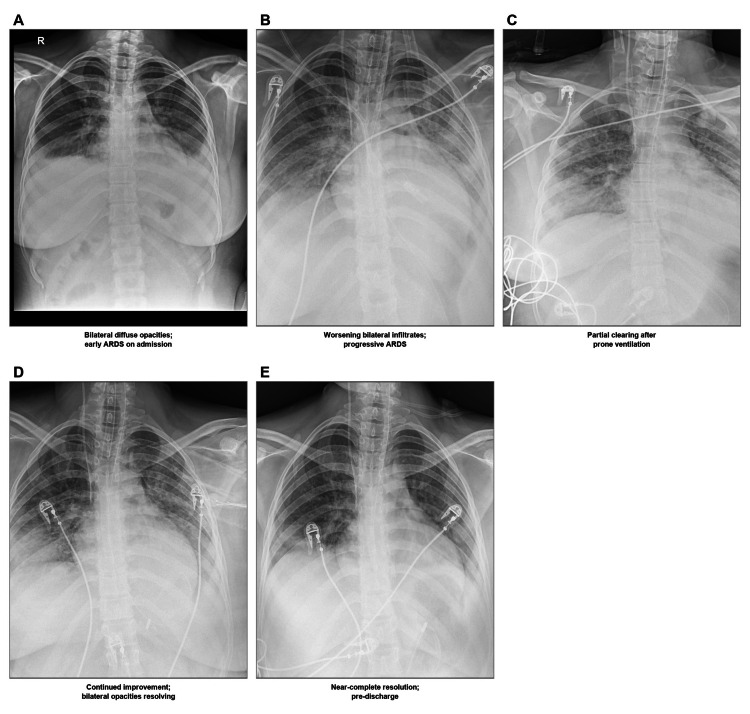
Serial anteroposterior chest X-rays demonstrating the progression and resolution of acute respiratory distress syndrome (ARDS) in a patient with concurrent Plasmodium falciparum malaria and dengue virus co-infection ARDS: acute respiratory distress syndrome; CCU: critical care unit; ECG: electrocardiography; ACPCV: assist-control pressure-controlled ventilation All images are portable anteroposterior chest X-rays. Day numbering is indexed to the first day of CCU admission. (A) Day 1: bilateral diffuse fluffy opacities consistent with early ARDS on admission to the CCU; endotracheal tube and bilateral ECG monitoring leads in situ. (B) Day 2: near-complete resolution of bilateral opacities; this X-ray was obtained prior to formal CCU admission and reflects a transient window of partial clinical stabilization before subsequent deterioration; it should not be interpreted as representing true radiological improvement in the context of the overall clinical trajectory. (C) Day 4: re-accumulation of bilateral patchy infiltrates with worsening consolidation consistent with severe ARDS, prompting initiation of prone ventilation; endotracheal tube, central venous catheter, and monitoring leads visible. (D) Day 5: partial clearing of bilateral infiltrates following an 18-hour prone ventilation session on Day 3, with persistent consolidation bilaterally; patient on ACPCV with ongoing lung-protective strategies. (E) Day 7: near-complete resolution of bilateral pulmonary infiltrates with bilateral clearing, corresponding to successful extubation on Day 6 and discharge from the CCU on Day 7

The patient's condition progressively deteriorated, necessitating critical care unit (CCU) admission. All subsequent "Day" references are indexed to Day 1 of CCU admission. On CCU admission (Day 1), she was conscious but irritable, febrile (101.9°F), tachycardic (pulse, 138 beats/minute), hypotensive (BP, 80/58 mmHg), and tachypneic (respiratory rate, 38 breaths/minute), with SpO₂ of 92% on 5 L/minute oxygen. Systemic examination revealed decreased air entry bilaterally with basal crepitations. Day 1 chest X-ray (CXR) demonstrated bilateral diffuse fluffy opacities consistent with early ARDS (Figure 1A). ECG demonstrated sinus tachycardia.

Laboratory investigations on CCU admission were as follows: hemoglobin, 8.2 g/dL; hematocrit, 24.6%; platelet count, 52,000/μLl total leukocyte count (TLC), 17,900/μL (neutrophils 69%, lymphocytes 27%); serum creatinine, 1.9 mg/dL; sodium, 132 mEq/L; potassium, 3.4 mEq/L; total bilirubin, 4.36 mg/dL; direct bilirubin, 2.64 mg/dL; ALT, 87 U/L; AST, 120 U/L; serum albumin, 2.7 g/dL; prothrombin time, 19.3 seconds; aPTT, 37 seconds; C-reactive protein (CRP), 14.04 mg/dL; random blood glucose, 198 mg/dL; and glucose-6-phosphate dehydrogenase (G6PD) activity within normal limits. Serology returned positive for dengue NS1 antigen and IgM antibody by ELISA. Scrub typhus, chikungunya, and leptospira serologies were all negative.

A two-dimensional echocardiogram demonstrated preserved left ventricular ejection fraction (LVEF, 60%) with a small pericardial effusion, effectively excluding cardiogenic pulmonary edema as the primary cause of respiratory failure. Abdominal ultrasonography showed hepatosplenomegaly. Arterial blood gas (ABG) analysis confirmed type 1 respiratory failure: pH, 7.41; PaO₂, 55.4 mmHg; PaCO₂, 28.2 mmHg; lactate, 3.5 mmol/L; base deficit, -2.3; and anion gap, 4.

The patient was resuscitated with intravenous crystalloid boluses and commenced on high-flow nasal oxygen (HFNO) at 40 L/minute. The ROX index (SpO₂/FiO₂ to respiratory rate ratio), calculated after one hour of HFNO, was 2.85, indicating a high likelihood of HFNO failure and the need for intubation [per established threshold <3.85]. She was intubated on Day 1 and initiated on invasive mechanical ventilation (assist-control pressure-controlled ventilation (ACPCV) mode) with concurrent noradrenaline support for vasoplegic shock.

Ventilatory strategy followed ARDS Network and European Society of Intensive Care Medicine (ESICM) lung-protective principles: tidal volume of 6 mL/kg predicted body weight (PBW), positive end-expiratory pressure (PEEP) titrated using the ARDSnet low PEEP/FiO₂ table, FiO₂ adjusted to maintain SpO₂ >94%, and plateau airway pressure maintained below 30 cmH₂O. Driving pressure was monitored where feasible. Analgosedation was provided with continuous fentanyl and midazolam infusions, titrated to a Richmond Agitation-Sedation Scale (RASS) score of -4.

By Day 4, the PaO₂/FiO₂ ratio had deteriorated to below 150 mmHg (PEEP ≥5 cmH₂O, FiO₂ 0.6), meeting the Berlin definition criteria for severe ARDS. The Day 4 CXR demonstrated worsening bilateral consolidation (Figure 1C). Prone positioning was initiated on Day 3 as a rescue strategy and maintained for 18 hours. Following prone ventilation, oxygenation improved progressively, with PaO₂/FiO₂ rising to 190-225 mmHg.

Fluid therapy throughout the CCU admission was strictly guided by the inferior vena cava distensibility index (dIVC) (threshold >18% indicating fluid responsiveness under fully controlled mechanical ventilation), complemented by serial lactate measurements, urine output, vasopressor trends, and meticulous intake-output charting to maintain a negative cumulative fluid balance from Day 4 onward (Table 1). It is acknowledged that dIVC has limitations in the context of low tidal volume ventilation, elevated PEEP, spontaneous respiratory effort, altered intra-abdominal pressure, and right ventricular dysfunction; it was therefore used as one component of a multimodal hemodynamic assessment rather than as a standalone guide.

**Table 1:** Daily clinical, hematological, biochemical, and ventilatory parameters across the seven-day CCU admission ACPCV: assist-control pressure-controlled ventilation; AKI: acute kidney injury; CCU: critical care unit; dIVC: inferior vena cava distensibility index; FiO₂: fraction of inspired oxygen; HFNO: high-flow nasal oxygen; MAP: mean arterial pressure; ND: not done/not measured; PaO₂/FiO₂: ratio of partial pressure of arterial oxygen to fraction of inspired oxygen; PBW: predicted body weight; PEEP: positive end-expiratory pressure; PSV: pressure support ventilation; SpO₂: peripheral oxygen saturation; TLC: total leucocyte count Day 1 refers to the first day of CCU admission. All subsequent days are indexed accordingly. ND: not done/not measured on that day. dIVC: IVC distensibility index = (Dmax − Dmin)/Dmin × 100; Dmax = maximal IVC diameter on inspiration, Dmin = minimal IVC diameter on expiration under fully controlled mechanical ventilation; dIVC >18% indicates fluid responsiveness. Net fluid balance = fluid intake minus urine output over 24 hours. Cumulative fluid balance = running total of net fluid balance from Day 1 of CCU admission. ARDS severity grading per Berlin definition: mild PaO₂/FiO₂, 200-300 mmHg; moderate, 100-200 mmHg; severe, <150 mmHg (on PEEP ≥5 cmH₂O). Noradrenaline dose expressed in μg/min; discontinued by Day 5. Tidal volume was maintained at 6 mL/kg PBW throughout ventilated days. Plateau airway pressure maintained below 30 cmH₂O; driving pressure (plateau pressure − PEEP) monitored with target below 15 cmH₂O where feasible

Parameter	Day 1	Day 2	Day 3	Day 4	Day 5	Day 6	Day 7	Reference value
pH	7.41	7.32	7.39	7.41	7.45	7.38	7.42	7.35-7.45
PaO₂/FiO₂ (mmHg)	150-180	144-165	90-170	190-225	230-250	280-325	350	>400
SpO₂ (%)	90-93	91-92	96-98	98-100	100	100	100	95-100
Lactate (mmol/L)	4.2	2.9	2.2	0.8	0.7	0.7	0.6	0.5-1.5
Hemoglobin (g/dL)	8.7	9.1	8.5	9.2	8.8	9.3	9.5	12–15
TLC (per cu mm)	ND	12,640	ND	9,870	ND	7,845	ND	4,000-11,000
Platelet count (per cu mm)	47,000	42,000	54,000	65,000	84,000	88,000	110,000	150,000-410,000
Bilirubin (mg/dL)	4.2	3.9	3.1	3.2	2.4	2.2	1.7	0.2-1.0
Creatinine (mg/dL)	2.2	2.3	1.9	1.7	1.4	1.1	1.3	0.5-1.5
MAP (mmHg)	55	60	68	70	72	71	69	70-100
dIVC (%)	24	22	18-22	15-17	14-16	10-12	11-12	12-18
Fluid intake (mL/24 h)	4,390	3,690	3,900	2,540	2,080	1,850	1,550	-
Urine output (mL/24 h)	1,505	1,620	2,330	3,770	2,410	1,975	2,800	-
Net fluid balance (mL/24 h)	+2,885	+2,070	+1,570	−1,230	−330	−125	−1,250	-
Cumulative fluid balance (mL)	+2,885	+4,955	+6,525	+5,295	+4,965	+4,840	+3,590	-
Noradrenaline (μg/min)	12-15	10-12	8-10	2-4	0	0	0	-
PEEP (cmH₂O)	8	8	10	10	8	-	-	-
FiO₂	0.6	0.6	0.7	0.6	0.4	-	-	-
Tidal volume (mL/kg PBW)	6	6	6	6	6	-	-	-
Respiratory support	ACPCV	ACPCV	Prone/ACPCV	ACPCV	PSV	HFNO	Oxygen	-

Regarding hemophagocytic lymphohistiocytosis (HLH), retrospectively, the combination of prolonged fever, bicytopenia, hepatosplenomegaly, and elevated inflammatory markers in this patient warrants acknowledgement that HLH workup, including serum ferritin, fibrinogen, triglycerides, and bone marrow examination, was not performed. HLH remains a differential to consider in similar future cases, particularly when cytopenias persist despite treatment.

Vasopressor support was weaned and discontinued by Days 4-5 as hemodynamics stabilized. The ventilator mode transitioned from ACPCV to pressure support ventilation (PSV) on Day 5, and the patient was successfully extubated on Day 6 with placement on HFNO and commencement of respiratory rehabilitation. The Day 7 CXR confirmed near-complete resolution of pulmonary infiltrates (Figure 1E). The patient was discharged from the CCU on Day 7 in a clinically stable condition.

## Discussion

Dengue and malaria are among the most common vector-borne febrile illnesses globally and are both highly endemic in Jharkhand. Because the two pathogens are transmitted by distinct mosquito genera, dengue by *Aedes aegypti *or *Aedes albopictus*, and malaria by *Anopheles species*, co-infection requires sequential bites from two different vectors within the respective incubation periods of each pathogen. In hyperendemic regions such as Jharkhand, where both *Aedes *and *Anopheles *populations co-exist with high transmission intensity, this is not uncommon. Importantly, no single mosquito species transmits both pathogens; co-infection is therefore always the result of two independent vector exposures.

Due to overlapping and nonspecific clinical presentations, dengue and malaria are frequently misdiagnosed as monoinfections rather than co-infections [6]. Our patient presented with features consistent with malaria-dengue co-infection, including fever, myalgia, vomiting, anemia, and thrombocytopenia. Dengue warning signs such as fever, vomiting, abdominal pain, and hepatomegaly have been reported to occur more frequently in co-infected patients in studies from the Brazilian Amazon [7]. Notably, hemoconcentration, a recognized dengue warning sign, was absent in our patient. The most plausible explanation is that malaria-induced anemia masked the hemoconcentration typically associated with dengue-related plasma leakage [8,9]. This phenomenon has been previously described in co-infected patients and further illustrates how concurrent malaria can attenuate or obscure classical dengue warning signs, compounding the diagnostic challenge. Our clinical findings mirrored those reported in that study.

Co-infection may amplify clinical severity. In a retrospective matched-pair study from French Guiana [10], co-infected patients demonstrated greater clinical severity, including deeper thrombocytopenia and more pronounced anemia, compared to monoinfections. A meta-analysis by Kotepui et al. [11] demonstrated that bleeding manifestations and jaundice are the most common complications. Studies from Bangladesh and Sub-Saharan Africa have similarly documented elevated rates of organ dysfunction in co-infected patients, reinforcing that this is a global phenomenon not limited to South Asia. Our patient developed jaundice and hepatosplenomegaly consistent with prior literature, but notably did not exhibit overt bleeding despite significant thrombocytopenia.

ARDS is a severe respiratory complication more commonly associated with *falciparum* malaria than dengue monoinfection [12,13]. Inflammatory lung injury has been implicated in malaria-associated ARDS [14], as evidenced by reports of noncardiogenic pulmonary edema even in *Plasmodium vivax* infection. In our patient, a synergistic inflammatory mechanism between the two pathogens likely contributed to the severity of ARDS, as demonstrated by the serial CXR progression from bilateral opacities on Day 1 to severe consolidation by Day 4, with gradual resolution following prone ventilation and lung-protective strategies (Figure 1).

ARDS was diagnosed using the Berlin definition criteria: bilateral pulmonary opacities not fully explained by effusions or atelectasis; onset within one week of the acute insult; PaO₂/FiO₂ <150 mmHg on PEEP ≥5 cmH₂O (severe ARDS); and exclusion of cardiogenic pulmonary edema supported by echocardiographic evidence of preserved LV function (LVEF 60%). Ventilatory management adhered to established evidence-based principles [15]: tidal volume 6 mL/kg PBW, PEEP/FiO₂ titration per ARDSnet protocol, plateau airway pressure below 30 cmH₂O, and driving pressure monitoring. Prone positioning for 18 hours on Day 3 was initiated per ESICM guidelines for severe ARDS (PaO₂/FiO₂ <150 mmHg) and resulted in meaningful oxygenation improvement.

Fluid management in dengue-malaria co-infection presents a fundamental challenge: adequate volume resuscitation is required for tissue perfusion during the early resuscitative phase, while subsequent volume restriction is critical to prevent worsening pulmonary edema in established ARDS. As reflected in Table 1, a net positive fluid balance was accepted in the first three days (peak cumulative +6,525 mL on Day 3), transitioning to de-resuscitation from Day 4 onward as hemodynamics stabilized and vasopressor requirements diminished [16,17]. The dIVC was used as one component of a multimodal assessment alongside serial lactate trends, urine output, and vasopressor requirements. We acknowledge dIVC limitations in low tidal volume ventilation, high PEEP states, and right ventricular dysfunction, and emphasize that clinical decisions were made through multiparameter integration rather than relying on dIVC in isolation.

Limitations

This is a single-case report; management recommendations cannot be generalized. Additional limitations include the following: HLH workup (ferritin, fibrinogen, triglycerides, bone marrow examination) was not performed; dengue serotyping was unavailable; pulmonary imaging relied on portable anteroposterior CXRs rather than CT; and formal driving pressure documentation was not available for all ventilator days. Future cases should consider HLH as a differential when cytopenias and systemic inflammation persist despite treatment of the primary infections.

## Conclusions

This case illustrates three core lessons. First, persistent fever and thrombocytopenia despite appropriate antimalarial therapy in a co-endemic region should prompt active investigation for dengue co-infection, avoiding the diagnostic anchoring that occurs when a first positive test ends the diagnostic process. Second, dengue-malaria co-infection can precipitate severe multiorgan dysfunction, including ARDS, hemodynamic shock, AKI, and hepatic injury, with a clinical trajectory potentially more severe than either monoinfection alone. Third, favorable outcomes in such complex co-infections are achievable through early intubation with strict lung-protective ventilation, early prone positioning in severe ARDS, and hemodynamically guided restrictive fluid management in the post-resuscitation phase.

These findings support routine dual pathogen screening, peripheral blood smear, and ELISA-based dengue serology, rather than defaulting to the first positive diagnosis, particularly in resource-limited tropical settings where co-endemicity is high. Strengthened surveillance programs are needed to capture the true burden of dengue-malaria co-infection, and future prospective multicenter studies are warranted to better define optimal management pathways.
